# Perfiles clínicos, bioquímicos y moleculares de tres neonatos de Sri Lanka con déficit de piruvato carboxilasa

**DOI:** 10.1515/almed-2024-0021

**Published:** 2024-05-15

**Authors:** Eresha Jasinge, Mihika Fernando, Neluwa-Liyanage Ruwan Indika, Pyara Dilani Ratnayake, Nalin Gamaathige, Ratnanathan Ratnaranjith, Sabine Schroeder, Patricia Jones, Skrahina Volha, Subhashinie Jayasena, Anusha Varuni Gunaratna, Asitha Niroshana Bandara Ekanayake, Arndt Rolfs

**Affiliations:** Departamento de Patología Química, Hospital Pediátrico Lady Ridgeway, Colombo 8, Sri Lanka; Departamento de Bioquímica, Facultad de Medicina, Universidad de Sri Jayewardenepura, Nugegoda, Sri Lanka; Departamento de Neurología, Hospital Pediátrico Lady Ridgeway, Colombo 8, Sri Lanka; Unidad de Cuidados Intesivos Neonatales, Hospital Maternal De Soysa Hospital, Colombo 8, Sri Lanka; Hospital General de Distrito, Vavuniya, Sri Lanka; CENTOGENE AG, Rostock, Alemania; Centro médico infantil, Centro médico Southwestern de la Universidad de Texas, Dallas, TX, EEUU; Universidad de Rostock, Rostock, Alemania

**Keywords:** genotipo, citrulina, neonato, fenotipo, déficit de piruvato carboxilasa, Introducción

## Abstract

**Objetivos:**

La piruvato carboxilasa (PC), una enzima mitocondrial, cataliza la conversión de piruvato, producto final de la glucólisis, en oxaloacetato, un intermediario del ciclo del ácido tricarboxílico. El déficit de piruvato carboxilasa, un trastorno raro, se manifiesta en tres fenotipos clínicos y bioquímicos: tipo de inicio neonatal (tipo A), tipo de inicio infantil (tipo B) y un tipo benigno (tipo C).

**Caso clínico:**

Presentamos el caso de tres neonatos de Sri Lanka, incluidos dos hermanos, de dos familias no emparentadas, con déficit de piruvato carboxilasa (DPC). Los tres desarrollaron dificultad respiratoria en las primeras horas de vida. Los dos hermanos presentaron las alteraciones bioquímicas típicas del tipo B. El tercer neonato presentaba niveles normales de citrulina y lisina, niveles moderados de lactato, lesiones quísticas paraventriculares, deformidades óseas y una nueva variante homocigótica sin sentido c.2746G>C [p.(Asp916His)] en el gen *PC*, indicativos bioquímicos de DPC tipo A.

**Conclusiones:**

Nuestros hallazgos indican la urgencia de realizar estudios analíticos en los neonatos que presenten taquipnea con acidosis metabólica concomitante, dado que la identificación temprana es crucial para el manejo del paciente y la prestación de consejo genético a la familia. Son necesarios más estudios para identificar los síntomas y alteraciones bioquímicas concurrentes en los diferentes fenotipos de déficit de piruvato carboxilasa.

## Introducción

La piruvato carboxilasa (PC, EC: 6.4.1.1) es una enzima mitocondrial que cataliza la carboxilación de piruvato en oxalacetato de forma dependiente de ATP [1]. PC, un homotetrámero de estructura tetraédrica dependiente de biotina [2], es activada alostéricamente por el acetil-CoA y está codificada por el gen localizado en el brazo largo del cromosoma 11 [3, 4]. PC es, principalmente, la responsable de la actividad anaplerótica, al reponer los intermediarios del ciclo del ácido tricarboxílico mediante oxaloacetato, además de ser una enzima reguladora de otras vías, como las de la gluconeogénesis, la lipogénesis, y las vías de producción de aminoácidos y neurotransmisores [5].

El déficit de piruvato carboxilasa (DPC; MIM# 266150), un trastorno autosómico recesivo con una incidencia estimada de 1 de cada 250.000 nacimientos [6], se manifiesta en tres fenotipos clínicos: El tipo A (tipo americano o forma infantil), que se suele manifestar algunos meses después del nacimiento en forma de retraso en el desarrollo, hipotonía, retraso en el crecimiento, y acidosis láctica leve o moderada, estando asociado a una mayor supervivencia. El tipo B (tipo francés o forma neonatal), tiene mal pronóstico, y se manifiesta principalmente durante las primeras 72 horas de vida en forma de taquipnea e hipotonía troncal grave [7, 8]. El tipo C (forma intermitente o benigna) se manifiesta durante el primer año de vida, con episodios de acidosis metabólica en situaciones de estrés fisiológico, con desarrollo intelectual normal o discapacidad intelectual (DI) leve [9, 10]. Las alteraciones bioquímicas, no siendo patognomónicas, pueden ayudar a diferenciar los tres fenotipos. Así, los pacientes con fenotipo de tipo B suelen presentar niveles elevados de lactato en plasma, a menudo por encima de los 10 mmol/L, y niveles elevados de citrulina y lisina, mientras que los pacientes con los tipos A y C suelen presentar niveles normales de citrulina [11]. El diagnóstico de DPC se establece en función de los niveles enzimáticos determinados en cultivos de fibroblastos de la piel, o linfoblastos, así como en la detección de variantes en el gen *PC* [11, 12]. La actividad enzimática residual no resulta útil a la hora de diferenciar los tres fenotipos principales, aunque influye en la gravedad de la presentación clínica [6, 7, 10]. La correlación entre el fenotipo y el genotipo muestra una elevada prevalencia de mutaciones con cambio de sentido en el tipo A, así como de mutaciones truncantes en el tipo B [11].

El objetivo principal de este estudio es describir los patrones bioquímicos de tres pacientes de Sri Lanka con DPC neonatal y señalar dos genotipos nunca antes descritos en un neonato con niveles normales de citrulina.

## Caso clínico

Seleccionamos a tres neonatos de dos familias no emparentadas de Sri Lanka (A y B) que fueron derivados en un periodo de siete años (2014–2020) al Servicio de Patología Química del Hospital Pediátrico Lady Ridgeway para la determinación de aminoácidos en plasma y ácidos orgánicos en orina. Los padres firmaron un consentimiento informado.

Los análisis bioquímicos de rutina en suero, así como el lactato plasmático se midieron en analizadores bioquímicos automáticos. Se recogieron muestras de plasma para aminoácidos en tubos EDTA, se centrifugaron inmediatamente, y se desproteinizaron y se analizaron mediante cromatografía líquida de intercambio iónico de alta resolución (HPLC) en el analizador de aminoácidos PerkinElmer, combinado con el sistema de derivatización post-columna con ninhidrina Pickering del Servicio de Patología Química del LRH Los reactivos, calibradores y muestras de control fueron adquiridos por Sigma Aldrich.

Los ácidos orgánicos se extrajeron aplicando métodos previamente optimizados [13] y se analizaron cualitativamente, empleando el sistema de cromatografía de gases combinado con espectrometría de masas (GC-MS) de Agilent. Todos los disolventes y demás reactivos eran de grado analítico.

La muestra de orina del paciente 2 de la familia A se conservó y transportó siguiendo las guías clínicas. Los aminoácidos se analizaron con el sistema Biochrome, mientras que los ácidos orgánicos se analizaron mediante GC-MS (Agilent) en el laboratorio del Hospital Pediátrico de Dallas de Texas (EE.UU), antes de la implementación de dichas pruebas al Servicio de Patología Química del LRH en 2017. Los aminoácidos en plasma y ácidos orgánicos en orina de los otros dos pacientes se analizaron en el Servicio de Patología Química del LRH.

Las muestras de sangre seca de los pacientes 2 y 3 se analizaron mediante cromatografía líquida acoplada a espectrometría de masas en tándem (LC-MS/MS) en NeoGen Labs Pvt. Ltd, India. El gen *PC* se analizó mediante secuenciación Sanger de los productos de reacción en cadena de la polimerasa obtenidos a partir de ADN de sangre periférica, en CENTOGENE AG (Alemania). La secuencia de referencia del gen *PC* es NM_000920.3.

### Familia A

**Figura 1:**
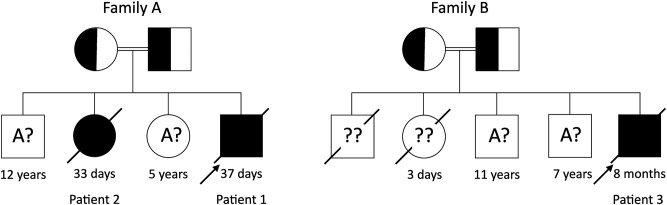
Árboles genealógicos de las Familias A y B. Las familias incluyen hermanos no afectados, así como hermanos enfermos cuyos genotipos exactos se desconocen (A? y ?? respectivamente). (A: alelo dominante, ?: alelo desconocido). Es probable que los hermanos que presentan la enfermedad sean homozigóticos (aa) para las variantes patogénicas. A: alelo dominante, a: alelo recesivo.?: alelo desconocido.

Los pacientes 1 y 2 son la descendencia de padres sanos consanguíneos procedentes de Sri Lanka, sin antecedentes médicos familiares de interés. El DPC se confirmó genéticamente en el paciente 1. Los padres tenían otros dos niños vivos sanos de 12 y 15 años, respectivamente. Los dos neonatos fallecieron a los 33 y 37 días de su nacimiento a causa de su patología (Figura 1).

#### Paciente 1

Un niño con un día de vida, el cuarto hijo de la familia, hijo de padres consanguíneos, nacido a término tras un embarazo sin complicaciones, con un peso al nacer de 2,190 kg (<−3SD) (Tabla 1). Había antecedentes de fallecimiento de un hermano a los 33 días de vida, con un diagnóstico bioquímico cuestionable de déficit de argininosuccinato sintasa.

**Tabla 1:** Características demográficas y clínicas.

	Paciente 1 [Familia A]	Paciente 2 [Familia A]	Paciente 3 [Familia B]
Edad de debut	Tres horas de vida	Al nacer	Una hora de vida
Sexo	Masculino	Femenino	Masculino
Consanguinidad	Sí	Sí	Sí
Hermanos afectados	Sí (paciente 2)	Sí (paciente 1)	Sí, dos muertes neonatales
Complicaciones del embarazo	Ninguna	Ninguna	Diabetes gestacional
Madurez al nacer	A término	A término	34 semanas
Tipo de parto	Vaginal	Vaginal	Cesárea
Peso al nacer, kg	2.19 (<−3SD)	2.05 (−2SD to −3SD)	2.05 (<−3SD)
Edad al morir	Día 37	Día 33	8 meses

Aunque no hubo complicaciones en el parto, el paciente desarrolló taquipnea a las tres horas de su nacimiento, precisando su ingreso en una Unidad de Cuidados Intensivos Neonatales (SCBU, por sus siglas en inglés). Tras examinarlo, el paciente presentaba cabello rubio, respiración acidótica, hepatomegalia leve e hipotonía generalizada, con reflejos preservados. La gasometría al ingreso reveló acidosis metabólica (pH 7,12, HCO_3–_ 4,2 mmol/L). La glucosa capilar era de 38 mg/dL, medida con un glucómetro portátil, siendo el lactato en plasma 18,75 mmol/L (0,5–2,2), con un resultado positivo para la presencia de cuerpos cetónicos en orina. El paciente sufrió convulsiones recurrentes en el hospital, que se manejaron eficazmente con tratamiento antiepiléptico con levetiracetam y fenobarbital. El análisis de aminoácidos en plasma (µmol/L) realizado al segundo día de vida mostró niveles elevados de citrulina, alanina, lisina y tirosina (Tabla 2). El perfil de ácidos orgánicos en orina reveló niveles muy altos de lactato, 3-hidroxibutirato (beta-hidroxibutirato), acetoacetato, 2-hidroxiburtirato, 4-hidroxifenillactato, concentraciones leves de 4-hidroxifenilpiruvato, fumárico y 2-hidroxiglutarato.

**Tabla 2:** Resultados de aminoácidos en µmol/L (sangre total, plasma y orina).

Aminoácido	Rangos de referencia			Paciente 1 [Familia A]	Paciente 2 [Familia A]		Paciente 3 [Familia B]	
	Plasma	DBS	Orina	Plasma	Orina	DBS	Plasma	DBS
Alanina	131–710	<960	767–6,090	**1,086**	323	241,19	448	456,2
Citrulina	3–55	3–75	22–181	**243**	**1,165**	**113,6**	17	23,7
Lisina	92–325	NP^a^	189–850	**861**	40	NR^a^	175	NR^a^
Tirosina	22–147	<300	20–1,650	**384**	62	214,94	85	81,1
Aspartato	20–129	9–108,1	0–240	**5**	2	14,25	18	44,4
Glutamato	92–325	19–265,83	55–590	**24**	**20**	39,08	70	48,76
Glutamina	200–1,200	58–450	393–1,562	**135**	**20**	54,99	253	246

Los valores alterados se indican en negrita. ^a^NR, no realizada.

Aunque debido a la presencia de niveles moderados de citrulina inicialmente se consideró la citrulinemia tipo 2 en el diagnóstico diferencial, los niveles elevados de alanina y los resultados alterados en las pruebas de función hepática (Tabla 2), nos llevaron a establecer el DPC como principal diagnóstico diferencial, por la grave acidosis láctica, y los niveles elevados de citrulina, alanina y lisina en plasma. El análisis genético del gen *PC* reveló una variante homocigótica sin sentido, probablemente una variante patogénica c.2514G>A [p.(Trp838*)], lo que confirmó el diagnóstico de DPC. Se optó por un manejo conservador con reemplazo de bicarbonato, vitamina B12, biotina, carnitina, coenzima Q, tiamina, piridoxina, aspartato y citrato de sodio/ácido cítrico (bicitra).

Tras un mes de tratamiento, y con los marcadores bioquímicos normalizados, el paciente fue dado de alta y continuó con la lactancia. Desgraciadamente, el bebé falleció a la semana del alta, posiblemente debido a la aspiración de leche.

Tras la confirmación genética de DPC, se revisó la historia de la hermana (paciente 2).

#### Paciente 2

Esta paciente nació a término mediante parto vaginal tras una gestación sin complicaciones, siendo la segunda hija de la familia A. Su peso al nacer fue de 2,05 kg (entre −2SD y −3SD). Desde el nacimiento, la recién nacida mostró poca actividad, rechazaba el alimento y presentó dificultad respiratoria. Al décimo día de su nacimiento, desarrolló acidosis grave (pH 7.21, HCO_3−_ 7,3 mmol/L) (Tabla 3) con cetonuria severa. El análisis de sangre seca mediante espectrometría de masas en tándem reveló niveles elevados de citrulina (113,6 μmol/L). El análisis de aminoácidos en orina mostró un aumento de citrulina (1.165 μmol/L) y ausencia de argininosuccinato (Tabla 2). El perfil de ácidos orgánicos en orina reveló cetosis severa (3-hidroxibutirato>acetoacetato) y excreción masiva de lactato, 3-fenillactato, 2-hidroxiisobutirato, 4-hidroxifenilacetato y 4-hidroxifenilpiruvato.

**Tabla 3:** Pruebas bioquímicas rutinarias realizadas al inicio de los síntomas.

Magnitud	Unidad	Rango de referencia	Paciente 1 [Familia A]	Paciente 2 [Familia A]	Paciente 3 [Familia B]
Lactato en plasma	mmol/L	0,5–2,3	**18,75**	NR^a^	**4–9,6**
Gasometría					
pH		7,33–7,44	**7,12**	**7,32**	**7,24**
HCO_3−_	mmol/L	23–28	**4,2**	**8,6**	**6,1**
Marcadores bioquímicos en suero					
Bilirrubina total	µmol/L	3–20	**61**	NR^a^	**134**
Bilirrubina directa	µmol/L	0–3	**27**	NR^a^	NR^a^
Fosfatasa alcalina	IU/L	60–425	**443**	NR^a^	153
Aspartato transaminasa	IU/L	0–40	**64**	**377**	**113**
Alanina transaminasa	IU/L	9–48	39	**301**	30
Gamma glutamil transferasa	IU/L	2–30	**211**	NR^a^	NR^a^
Proteína C-reactiva	mg/L	< 5,0	34	23	NR^a^
Creatina quinasa	IU/L	28–300	**1,228**	NR^a^	181
Urea	mmol/L	1,0–2,5	**7,9**	**4**	**3,9**
Creatinina	µmol/L	35–40	**75**	16	**45**
Colesterol total	mmol/L	1,09–2,07	**2,58**	NR^a^	1,69
Triglicéridos	mmol/L	0,97–3,13	**4,5**	NR^a^	0,95

Los niveles elevados de lactato (y de ahí, la acidosis metabólica) y los niveles de lípidos son secundario a la acumulación de piruvato y acetil-CoA, respectivamente. Los niveles elevados de bilirrubina y de enzimas hepáticas indican disfunción hepatocelular. Los valores alterados se indican en negrita. ^a^NR, no realizada.

El diagnóstico de déficit de argininosuccinato sintasa se estableció en base a los resultados de los perfiles de aminoácidos en sangre y orina. La confirmación genética no era viable en aquel momento. Se trató a la paciente con fluidoterapia intravenosa y bicarbonato en la SCBU. A los 21 de su nacimiento, presentaba un peso de 1,81 kg (<−3SD). A los 33 días, la paciente falleció a causa de su patología. En el análisis retrospectivo del perfil clínico y bioquímico de un neonato con dificultad respiratoria, acidosis metabólica severa, niveles elevados de citrulina y aciduria láctica, y considerando los resultados genéticos del hermano (paciente 1), también se estableció el diagnóstico de DPC en la hermana.

### Familia B

El paciente 3 era hijo de padres consanguíneas procedentes de Sri Lanka. Los primeros dos hijos, un niño y una niña, fallecieron al tercer día de vida a causa de una dificultad respiratoria. La familia tiene dos hijos sanos vivos de 11 y 7 años, respectivamente (Figura 1).

#### Paciente 3

Se trataba del quinto hijo de la pareja y nació mediante cesárea de urgencia a las 33+3 semanas de gestación, debido a que la madre había dado a luz por cesárea anteriormente. Excepto por la diabetes gestacional de la madre, de 39 años de edad, no hubo complicaciones durante el embarazo.

Sus puntuaciones Apgar a los 1, 5 y 10 minutos fueron de 9-10-10 respectivamente, con un peso al nacer de 2,05 kg (<−3SD), una longitud de 48 cm (−1SD) y una circunferencia craneal de 33 cm (−1SD con respecto a la mediana). Dado que el neonato presentó dificultad respiratoria y taquipnea en su primera hora de vida, fue ingresado en la SCBU. La gasometría a las 3 horas de vida reveló acidosis metabólica (pH 7,27, pCO_2_ 42 mmHg, HCO_3−_ 18,3 mmol/L, lactato 4 mmol/L). Se trató al paciente con bicarbonato intravenoso y 2 L de oxígeno mediante cánula nasal. La segunda gasometría realizada a las 7 h de vida indicó una mejoría de los parámetros, excepto en los niveles de lactato, que se mantenían elevados (pH 7,41, pCO_2_ 26,8, pO_2_ 66, HCO_3−_ 16,8, lactato 4,6). Al cuarto día de vida, se produjo una mejoría clínica, siendo trasladado el bebé a la planta de neonatos, para mantenerlo en observación. Así mismo, el examen neonatal reveló una deformidad en la pierna derecha (hemimelia del peroné con sindesmosis del segundo dedo del pie con pie subdesarrollado) y suturas craneales superpuestas.

Al séptimo día de su nacimiento, el paciente fue ingresado nuevamente en la SBCU, debido a una disminución de la actividad, mala succión, dificultad respiratoria y problemas en la ganancia de peso (pérdida de peso del 12 %). Se trató un episodio de hipoglucemia al ingreso (el nivel de glucosa en sangre capilar era de 36 mg%) con un bolo intravenoso de dextrosa al 10 %. Dado que la acidosis metabólica persistía (pH 7,24, pCO_2_ 6, lactato 4,6, HCO_3−_ 6,1) se realizó corrección con bicarbonato. Las siguientes gasometrías mostraron una mejoría del pH, así como de los niveles de bicarbonato, aunque se mantenían los niveles elevados de lactato (9,6 mmol/L). El análisis de aminoácidos en plasma realizado al octavo día de vida mostró niveles normales de citrulina, alanina y lisina. El análisis simultáneo de la muestra de DBS mediante espectrometría de masas en tándem mostró un perfil de aminoácidos normal (Tabla 2). El perfil de ácidos orgánicos en orina mostró niveles muy elevados de lactato, y una elevación leve de 4-hidroxifenillactato, 3-hidroxibutirato, acetoacetato, fumarato y 2-hidroxiglutarato. Se reinició la oxigenoterapia con cánula nasal y el tratamiento antibiótico. Se administró corrección con bicarbonato sódico intravenoso tres veces y, posteriormente, se cambió a bicarbonato sódico oral (2 mmol/kg/día dividido en dos dosis). A los tres días del reingreso, el bebé experimentó una mejoría clínica, aunque persistían los niveles elevados de lactato y la acidosis metabólica. El neumotórax leve en el pulmón derecho observado en la radiografía se resolvió de manera espontánea.

Al alta a los 19 días del nacimiento, el paciente se encontraba clínica y bioquímicamente estable, aunque el lactato permanecía ligeramente elevado (pH 7,4, pCO_2_ 30,3, pO_2_ 71, HCO_3−_ 22,3, lactato 3,1). Tras disminuir su peso a 1,78 kg, un fenómeno fisiológico, lo recuperó a un ritmo de 15 g/día, hasta alcanzar un peso de 1,86 kg al alta. Se le alimentó con leche materna y de fórmula con suplementos de hierro y multivitamínicos.

Se le realizó un seguimiento periódico en una clínica pediátrica y ortopédica. A los 2 meses de edad, el paciente pesaba 2,8 kg (−2SD to −3SD), presentaba una altura de 53 cm (−1SD to −2SD) y una circunferencia craneal de 37 cm (+1SD to +2SD). La ecografía craneal, realizada como parte del seguimiento habitual en los bebés prematuros, reveló quistes porencefálicos en el lóbulo frontal adyacente al cuerno anterior, un quiste en el lado derecho de 12,5×15,1 mm, y un quiste en el lado izquierdo de 12,5×17,2 mm, aunque la fosa posterior y los ventrículos bilaterales eran normales.

A los 6 meses de edad, su retraso en el desarrollo era evidente y la antropometría revelaba una escasa ganancia de peso [6,8 kg (−1SD to −2SD)], sin mejoría en el crecimiento [65 cm (−2SD to −3SD)] y una notable microcefalia de 41 cm (−2SD to −3SD).

Se identificó la variante de cambio de sentido en homocigosis c.2746G>C [p.(Asp916His)] en el gen *PC*, estableciéndose el diagnóstico de DPC. La variante se clasificó como de significado incierto, siguiendo las recomendaciones de CENTOGENE y del Colegio Americano de Genética y Genómica Médica (ACMG).

A los ocho meses de edad, el paciente desarrolló una infección de las vías respiratorias inferiores, lo que derivó en acidosis metabólica refractaria grave. El bebé falleció a pesar de su ingreso en cuidados intensivos en un hospital terciario.

## Discusión

Describimos los perfiles clínicos, bioquímicos y moleculares de tres neonatos de Sri Lanka con déficit de piruvato carboxilasa. La presentación demográfica y clínica de los tres pacientes parece homogénea, ya que los tres eran hijos de padres consanguíneos con hermanos afectados, bajo peso al nacer y desarrollo temprano de dificultad respiratoria.

El retraso en el crecimiento prenatal, que se manifiesta en un bajo peso al nacer y se observó en los tres neonatos, es un hallazgo probable en los defectos del metabolismo energético neonatal, aunque se han descrito casos de pacientes con un peso normal al nacer [14, 15]. Las deformidades óseas observadas en el paciente 3, un hallazgo nunca antes descrito, podría ser una alteración puntual, aunque debería ser objeto de estudio.

Nuestros pacientes presentaban las alteraciones bioquímicas descritas en la literatura, como acidosis metabólica y elevación del lactato en plasma [14, 15] y las transaminasas [14]. Además, el paciente 1 mostró una leve elevación de la bilirrubina directa, la fosfatasa alcalina y la gamma glutamil transferasa. La leve elevación del colesterol en dicho paciente podría deberse a la desviación del exceso de cuerpos cetónicos para producir acetil CoA y acetoacetil CoA, tal como se describe en el DPC tipos A y B [16].

La hipoglicemia observada en los pacientes 1 y 3, un hallazgo esperable, dado que la PC participa directamente en la gluconeogénesis [11], se ha descrito en la literatura [14]. Las alteraciones en el perfil de aminoácidos del paciente 1 estaban claramente relacionadas con la DPC. Por ejemplo, la elevación de la citrulina se debe a la deficiencia de oxaloacetato y, por tanto, del aspartato, una aminoácido necesario para sintetizar argininosuccinato a partir de la citrulina. Esta alteración se suele observar en pacientes con DPC tipo B [11] y se detectó en el análisis de DBS, plasma u orina de los dos hermanos. La elevación de lisina y los niveles bajos de glutamato (por lo tanto, glutamina baja) en el paciente 1 pueden explicarse por la deficiencia de alfa-cetoglutarato (2-oxoglutarato), implicado en la descomposición de la lisina y la producción de glutamato. Los niveles de alanina pueden aumentar por la transaminación del piruvato acumulado. Los resultados de ácidos orgánicos urinarios (elevación del lactato, el acetoacetato, y el 3-hidroxibutirato), sumado a la hipoglucemia, orientaron la sospecha diagnóstica hacia DPC. Sin embargo, el déficit de argininosuccinato sintasa (ASSD) se caracteriza por niveles elevados de amoníaco, glutamina y citrulina [17], por lo que los niveles bajos de glutamina en el paciente 1 descartaban en gran medida un diagnóstico de ASSD. La hiperamonemia es también un fenotipo metabólico de la DPC tipo B [18]. Por el contrario, la elevación de la tirosina que presentaba el paciente 1 no se suele observar en la DPC tipo B [19].

**Figura 2:**
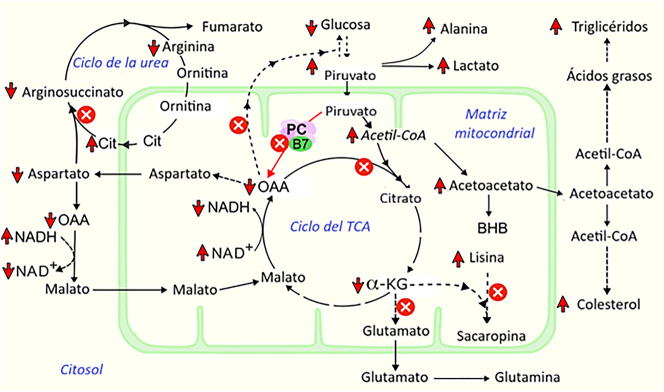
Cambios bioquímicos asociados al déficit de piruvato carboxilasa; Los intermediarios del ciclo del ácido tricarboxílico (TCA) disminuyen. La gluconeogénesis se ve afectada debido a la reducción de la disponibilidad de oxalato. El ciclo de la urea se altera debido a la deficiencia de aspartato, lo que resulta en hipercitrulinemia. La degradación de la lisina puede verse afectada debido a la deficiencia de alfa-cetoglutarato. El aumento de acetil-CoA se dirige a la cetogénesis, la síntesis de ácidos grasos y la síntesis de colesterol. La relación NAD^+^/NADH citoplasmática disminuye, mientras que la relación NAD^+^/NADH mitocondrial aumenta. α-KG, alfa-cetoglutarato; B7, biotina; BHB, beta-hidroxibutirato; Cit, citrulina; HMG-CoA, β-hidroxi- β-metilglutaril-CoA; NAD^+^, nicotinamida adenina dinucleótido oxidado; NADH, nicotinamida adenina dinucleótido reducido; OAA, oxaloacetato; PC, piruvato carboxilasa.

Las manifestaciones bioquímicas y clínicas nos hicieron sospechar que ambos padecían de una DPC tipo B. En un principio, al paciente 2 se le diagnosticó ASSD, en base únicamente a la elevación de la citrulina en sangre total y en orina, en ausencia de argininosuccinato, a pesar de que los niveles de citrulina en sangre total no se encontraban en el rango esperado en la ASSD. El diagnóstico de DPC se realizó retrospectivamente, tras establecer el diagnóstico del hermano (paciente 1). Este caso ilustra las dificultades a la hora de establecer un diagnóstico, cuando no se dispone de las pruebas necesarias para ello, entre las que se encuentran la determinación del lactato, una importante prueba que, sin embargo, no se realiza en la mayoría de los laboratorios de Sri Lanka. La elevación del lactato es característica de la DPC [6, 14]. En la Figura 2 se muestran las alteraciones bioquímicas asociadas a la DPC.

En el paciente 3, la espectrometría de masas en tándem en sangre total y la cromatografía líquida de alto rendimiento en plasma revelaron niveles normales de citrulina. Aunque el debut neonatal nos inclina a pensar en DPC tipo B, el lactato en plasma (<10 mmol/L) y los niveles normales de citrulina sugieren DPC tipo A. En la literatura, se ha descrito un caso de DPC tipo A en un neonato con niveles normales de citrulina y lisina [20].

Los niveles elevados de lactato, 4-hidroxifenilacetato y 4-hidroxifenillactato fueron un hallazgo constante en los perfiles de ácidos orgánicos en orina de los tres pacientes. Este hallazgo concuerda con un caso clínico publicado recientemente, en el que se describían niveles elevados de 4-hidroxifenilacetato y 4-hidroxifenillactato en orina [19]. Esto podría indicar una inhibición secundaria en el catabolismo de la tirosina. Así mismo, los niveles de los compuestos cetónicos fueron moderados, con niveles bajos de compuestos derivados de ácidos tricarboxílicos. La mayoría de los datos publicados indican niveles elevados de 2-hidroxibutirato, 3-hidroxibutirato y acetoacetato en orina, y niveles reducidos de compuestos derivados de ácido tricarboxílico, como alfa-cetoglutarato (2-oxoglutarato), fumarato, oxaloacetato y malato en orina [11, 14].

Los pacientes con DPC tipo B también presentan una elevación de piruvato, relación lactato/piruvato y relación acetato/3-hidroxibutirato en sangre [18]. Sin embargo, en Sri Lanka no se realizan las pruebas de piruvato, acetoacetato o 3-hidroxibutirato en plasma, por lo que estas relaciones no se pudieron calcular en nuestros pacientes.

Los tres casos descritos mostraron una serie de alteraciones bioquímicas generales, como niveles séricos elevados de bilirrubina, aspartato transaminasa, fosfatasa alcalina y creatina fosfato. Estas alteraciones coinciden con las descritas en un caso clínico de un neonato con DPC tipo B [19].

Las cavidades paraventriculares simétricas en torno a los cuernos frontal y temporal de los ventrículos laterales observadas en el paciente 3 han sido descritas anteriormente en la literatura [21, 22]. Las alteraciones de la lipogénesis debido a la deficiencia de oxaloacetato citosólico podrían explicar este hallazgo, así como la significativa desmielinización de la materia blanca cerebral y cerebelar.

Aunque los dos hermanos parecen ser portadores de la misma mutación en homocigosis, presentaban alteraciones bioquímicas distintas. En la literatura, las mutaciones con cambio de sentido complejas, las mutaciones en el sitio de empalme, la deleciones en homocigosis, así como heterocigosis compuesta y mosaicismo, se han descrito como fenotipos de DPC tipo B. Por otro lado, los pacientes con DPC tipo A suelen ser portadores de mutaciones con cambio de sentido en homocigosis o en heterocigosis compuesta [11, 15, 18, 23]. Se desconoce el mecanismo de esta variabilidad bioquímica y genética, que podría ser multifactorial y depender de la cantidad de proteína PC, el nivel de actividad enzimática residual en los tejidos, las variantes genéticas y la influencia de factores ambientales [23].

La variante PC c.2514G>A p.(Trp838*) del paciente 1, y presumiblemente de la paciente 2, que crea un codón de parada, explica la gravedad del fenotipo, en contraste con la nueva variante de cambio de sentido c.2746G>C p.(Asp916His) que, en el paciente 3, provocó un cambio de aminoácido de aspartato a histidina en la posición 916. Las alteraciones clínicas y bioquímicas observadas en los dos neonatos de la familia A concuerdan con DPC tipo B, mientras que las alteraciones bioquímicas y moleculares del paciente 3, aunque el debut fuera neonatal, eran más indicativas del tipo A.

Este estudio confirma la presentación y resultados clínicos descritos en la literatura, aunque algunas alteraciones no habían sido descritas anteriormente. Los resultados de este estudio concienciarán a los clínicos sobre la existencia de este raro trastorno, así como de la necesidad de realizar estudios más amplios.

### Lecciones aprendidas

– El lactato en plasma, el perfil de aminoácidos en plasma y el perfil de ácidos orgánicos resultan útiles a la hora de establecer un diagnóstico provisional de deficiencia de piruvato carboxilasa en los países en vías de desarrollo.
– La elevación sérica de aspartato transaminasa, fosfatasa alcalina y creatina fosfato podrían ser alteraciones bioquímicas asociadas al déficit de piruvato carboxilasa tipo B.
– Los niveles de 4-hidroxifenilpiruvato y 4-hidroxifenillactato en orina permanecieron constantemente elevados en los tres casos.
